# Transcriptional Analyses of Mandarins Seriously Infected by ‘*Candidatus* Liberibacter asiaticus’

**DOI:** 10.1371/journal.pone.0133652

**Published:** 2015-07-21

**Authors:** Meirong Xu, Ya Li, Zheng Zheng, Zehan Dai, Yang Tao, Xiaoling Deng

**Affiliations:** Citrus Huanglongbing Research Laboratory, South China Agricultural University, Guangzhou, Guangdong, China; Key Laboratory of Horticultural Plant Biology (MOE), CHINA

## Abstract

A range of leaf symptoms, including blotchy mottle, yellowing, and small, upright leaves with a variety of chlorotic patterns resembling those induced by zinc deficiencies, are associated with huanglongbing (HLB, yellow shoot disease), a worldwide destructive citrus disease. HLB is presumably caused by the phloem-limited fastidious prokaryotic α-proteobacterium ‘*Candidatus* Liberibacter spp.’ Previous studies focused on the proteome and transcriptome analyses of citrus 5 to 35 weeks after ‘*Ca*. L. spp.’ inoculation. In this study, gene expression profiles were analyzed from mandarin *Citrus reticulate* Blanco cv. jiaogan leaves after a 2 year infection with ‘*Ca*. L. asiaticus’. The Affymetrix microarray analysis explored 2,017 differentially expressed genes. Of the 1,364 genes had known functions, 938 (46.5%) were up-regulated. Genes related to photosynthesis, carbohydrate metabolic, and structure were mostly down-regulated, with rates of 92.7%, 61.0%, and 80.2%, respectively. Genes associated with oxidation-reduction and transport were mostly up-regulated with the rates of 75.0% and 64.6%, respectively. Our data analyses implied that the infection of ‘*Ca*. L. asiaticus’ could alter hormone crosstalk, inducing the jasmine acid pathway and depressing the ethylene and salicylic acid pathways in the citrus host. This study provides an enhanced insight into the host response of citrus to ‘*Ca*. L. asiaticus’ infection at a two-years infection stage.

## Introduction

Citrus in tropical and sub-tropical areas is subjected to Huanglongbing (HLB, Yellow shoot disease), a highly descructive bacterial disease. Whole genome databases of two citrus varieties have been constructed (http://citrus.pw.usda.gov/, http://www.citrusgenomedb.org and http://citrus.hzau.edu.cn/orange/download/index.php) [1, 2]. Substantial research efforts have been made to study the citrus-’*Candidatus* Liberibacter spp.’ interaction using microarrays based on expression sequence tags, hi-seq based on transcriptome and comparative proteomic approaches [3–13] (Table 1).

**Table 1:** A list of citrus genomic, transcriptomic or proteomic studies.

studies	Plant materials	replications	Method	Time after inoculation	Ref.	No. of differentially expressed genes	
						Up-regulated	Down-regulated
1	3-yr Valencia sweet orange, leaf	3 or 2	Microarray	5–9 WAI	[3]	158	22
2	3-yr Valencia sweet orange, leaf	3 or 2	Microarray	13–17 WAI	[3]	394	285
3	Young sweet orange, leaf	3	Microarray	8 MAI	[5]	307	317
4	2-yr Madam Vinous, leaf	3	iTRAQ	7 MAI	[4]	10 (SY) and 13 (AS)	7 (AS)
5	2-yr Madam Vinous, leaf	3	Microarray	7 MAI	[4]	1953	1523
6	15-mo Cleopatra, leaf		Microarray	30–32 WAI	[6]	326 (>4) 1110	626
7	15-mo US-897 (Poncirus * Blanco), leaf		Microarray	30–32 WAI	[6]	17 (>4); 261	39
8	Valencia sweet orange, fruit		RNA-seq	Unknown	[7]	925(SY vs H); 617(AS vs H); 437(AH vs H); 223 (AH vs AS); 288(SY vs AS); 516(SY vs AH)	783(SY vs H); 550(AS vs H); 689(AH vs H); 70(AH vs AS); 271(SY vs AS); 248(SY vs AH)
9	18-yr Hamlin, fruit; 18-yr Valencia, fruit-FF, VT, and JV		Microarray	Unknown	[8]	Hamlin (SY/H): 174(JV), 397(VT), 679(FF); Valencia (SY/H): 291(JV), 288(VT), 209(FF); Valencia (AS/H): 16(JV), 168(VT), 269(FF); Valencia (S/AS): 233(JV), 110(VT), 63(FF)	Hamlin (SY/H): 111(JV), 466(VT), 700(FF); Valencia (SY/H): 105(JV), 252(VT), 302(FF); Valencia (AS/H): 12(JV), 232(VT), 366(FF); Valencia (S/AS): 106(JV), 57(VT), 50(FF)
10	2-yr-grapefruit, leaf		2-DE	3 MAI	[9]	16(IP vs UP); 92(IS vs US); 12(US vs UP); 17(IS vs IP)	11(IP vs UP); 71(IS vs US); 18(US vs UP); 70(IS vs IP)
12	Lemon, leaf	2	2-DE	6 MAI	[10]	10	24
11	Sweet orange Pera and Hamlin	2	Microarray	>32 WAI	[11]	418	215
12	2-yr-Valencia sweet orange, stem	3	Microarray	16 MAI	[12]	551	334
13	2-yr-Valencia sweet orange, root	3	Microarray	16 MAI	[12]	56	55
14	‘Valencia’ sweet orange in the field, leaf (YL and ML) and fruit (IF and MF)		RNA-Seq	Unknown	[13]		

Yr, year; mo, month; SY, symptomatic; AS, asymptomatic; AH: apparently healthy; H, healthy; FF, flavedo; VT, vascular tissue; and JV, juice vesicles; YL, immature leaves; ML, mature leaves; IF, immature fruit peel; MF, mature fruit peel; IP, infected pre-symptomatic; UP, the uninfected pre-symptomatic; US, uninfected control for symptomatic; IS, infected symptomatic.

The use of genomic, transcriptomic, and proteomic approaches in the study of citrus responses to ‘*Ca*. L. spp.’ infections has improved our knowledge on HLB. The microarray method has been used to study the gene expression changes in different citrus leaves or fruits. Differentially expressed genes (DEGs) of ‘*Ca*. L. asiaticus infected ‘Valencia’ oranges involved in cell defense, transport, cellular organization, photosynthesis, and carbohydrate metabolism were most notable, especially the genes coding the phloem-specific lectin PP2-like protein and starch biosynthetic genes [3]. The isobaric tags for relative and absolute quantitation (iTRAQ) technique was used to characterize proteome changes in ‘Madam Vinous’ after ‘*Ca*. L. asiaticus’ inoculation, identifying potential targets of early infections, such as four miraculin-like proteins, chitinase, Cu/Zn superoxide dismutase and lipoxygenase [4]. The RNA-seq method has been used to characterize the gene regulatory network in ‘Valencia’ sweet orange’s response to ‘*Ca*. L. asiaticus’[7, 13]. The pathogen-induced accumulation of transcripts for phloem-specific lectin PP2-like protein, ADP-glucose pyrophosphorylase, starch synthase, granule-bound starch synthase, starch debranching enzyme, miraculin-like proteins, Cu/Zn superoxide dismutase, 2-oxoglutarate and Fe(II)-dependent oxygenases, WRKY transcription factors, sugar transporters, chitinases, lectin-related proteins, peroxiredoxins and a CAP 160 protein, were notable. The ‘*Ca*. L. asiaticus’ infection triggered a decrease in the production of photosynthesis-related proteins, defense-related pathogen-response proteins, oxygen-evolving enhancer proteins, a PSI 9 kDa protein and a Btf3-like protein were notable.

Most studies mentioned in Table 1 used leaf materials, including symptomatic (SY), asymptomatic (AS), apparently healthy (AH), healthy (H), immature leaves (IL) and mature leaves (ML). RNA samples of fruit, including flavedo, peel, vascular tissue, or juice vesicles, roots and stems were also extracted for the high-throughput analysis of citrus responding to ‘*Ca*. L. asiaticus’ infection (Table 1). The leaf, stem and root samples from the individual studies were collected within one year of ‘*Ca*. L. asiaticus’ infection. Moreover, fruit samples were collected from trees with an ambiguous infection time, probably more than 12 months after infection. In the citrus industry, the growers generally keep the diseased trees until almost no economic value is output, usually more than 2 years.

The objective of this study was to identify, the groups of citrus genes involved in the late stage of ‘*Ca*. L. asiaticus’ infections to provide information for HLB management.

## Materials and Methods

### Plant materials

**Fig 1:**
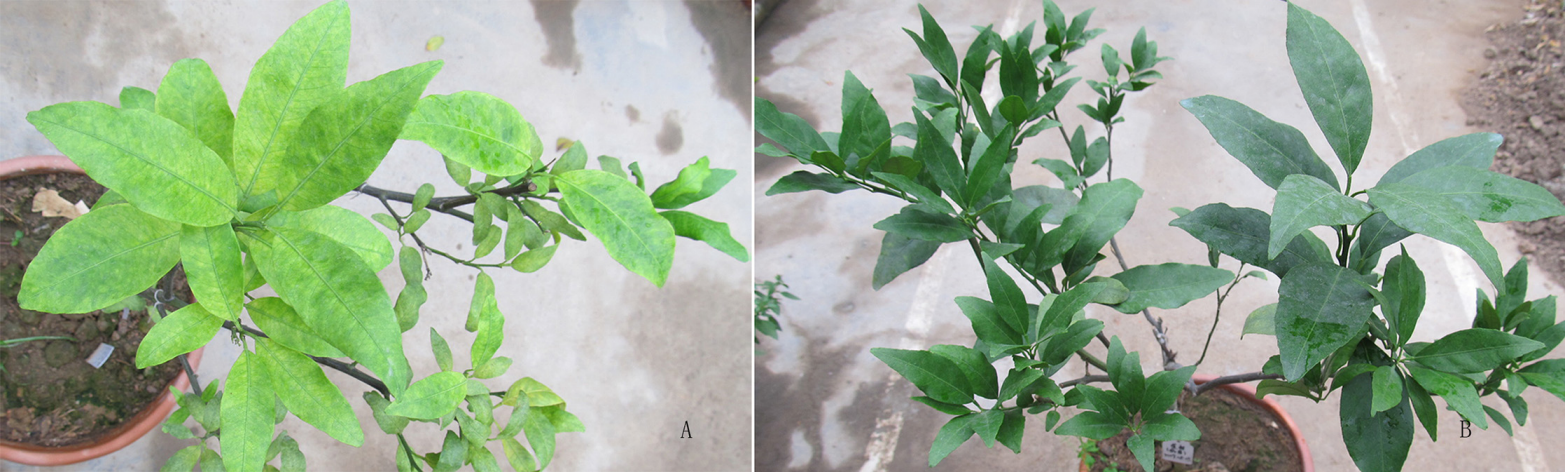
Phenotype symptoms of *Citrus reticulata* Blanco cv. Jiaogan 25 month-after-inoculation. A, One of the three trees grafted with diseased scion; B, one of the three trees grafted with healthy scion.

These experiments were performed using *Citrus reticulate* Blanco cv. jiaogan in 2012. The Affymetrix GeneChip citrus genome were applied to study the molecular pathways mediated by ‘*Ca*. L. asiaticus’ inoculated 3-year-old jiaogan seedlings. Ten jiaogan trees scioned on Cleopatra mandarin (*Citrus reticulata* Blanco) rootstocks kept in screenhouse, under natural light conditions were selected. Each of them was graft-inoculated with one sweet orange scion with or without ‘*Ca*. L. asiaticus’ in Dectember, 2009. Two years later, PCR detection of ‘*Ca*. L. asiaticus’ using primer pairs OI1/OI2c [14] identified 5 positive trees with typical blotchy mottling symptoms, while the other 5 with healthy leaves were confirmed negative for *Ca*. L. asiaticus. Of them, three trees showing similar HLB symptoms and similar size were chosen as materials (Fig 1A). Three healthy trees without ‘*Ca*. L. asiaticus’ infection with similar growth conditions were selected as controls (Fig 1B). Fully expanded mature leaves with blotchy symptoms and leaves at the similar portions on healthy trees were collected for RNA extraction at 25 month after inoculation.

### DNA extraction and conventional PCR detection

About 0.1 g midribs of leaves were cut into pieces and mixed with CXD buffer provided by HP plant DNA kit (D2485-02, OMEGA bio-tek). Samples were pulverized using MP FastPrep-24 machine, and DNA was extracted according to manufacturer’s instruction. The final DNA pellets were dissolved in 100 μL elution buffer and checked by electrophoresis in 1% (w/v) agarose gel. The concentration and quality of DNA were determined with an Agilent Model 2100 Bioanalyzer (Agilent Technologies, Palo Alto, CA), and all samples were adjusted to a final concentration of 50 ng/μL. Detection and identification of the ‘*Ca*. L. asiaticus’ was based on primer pairs OI1/OI2c [14].

### RNA extraction, array analysis, and digital gene expression

Around 0.5 g of leaves grounded by mortar and pestle in liquid nitrogen was used for total RNA extraction. RNA manipulation was done according to SDS method with few modifications. Leaf samples were ground in 0.7 mL of extraction buffer mixed with 2% SDS, 0.02 mol/L Borax, 4% β- Mercaptoethanol, 3% Tris-phenol, and 2.2% Chloroform. Fine tissue was vortex mixed for 3 min at 6,000 rpm, centrifuged at 12,000 rpm for 5 min (4°C). The aqueous phase was transferred to a new tube, and then 0.6 mL of Tris-phenol and Chloroform were added separately, followed by 2 min of vortex and 5 min of spin respectively. The RNA was precipitated from the aqueous phase by mixing with 4M LiCl and ethanol. Diluted nucleic acid was treated with 3M NaAc and Ethanol on ice for 10 min. The RNA pellet was washed once with 75% Ethanol (in DEPC-treated water), inverted, mixed and centrifuged at 12,000 rpm for 30 minutes at 4°C. The RNA pellet was dried at room temperature and dissolved in 60 μL RNase-free water by passing the solution through the pipette tip for a few times. All RNA samples was qualified by Agilent Model 2100 Bioanalyzer and kept in -70°C for further use.

Real time PCR of 25 citrus genes (mentioned in [15]) reported to be differentially expressed in the previous microarray experiment [3–6] were carefully carried out using the 6 samples collected here to test the gene expression consistency within biological repeats. No significant difference was observed among the ‘*Ca*. L.asiaticus’ treated or non-treated samples for all 25 genes including 2 housekeeping candidates. Consequently, the same amounts (ng) of three infected RNA samples or healthy samples were mixed respectively. Mixed RNA samples were sent to a biological company (CapitalBio corperation, Beijing) for cDNA preparation, hybridization, scanning, and image analysis of the arrays according to the manufacturer’s recommendations (Affymetrix, Santa Clara, CA, USA). The microarray hybridization was based on 33,879 expressed sequence tag sequences from different citrus (http://www.affymetrix.com/products/arrays/specific/citrus.affx). Differentially expressed genes were screened with a cutoff threshold P value of 0.05 and log_2_ fold change (LFC) of≧2.00 or ≦-2.00.

### Reverse transcription and quantitative RT-PCR

Three μg RNA of each sample was reverse transcribed using ReverTra Ace qPCR RT Kit (TOYOBO, Code No.: FSQ-101). The cDNA samples were quality-checked and quantified using Agilent Model 2100 Bioanalyzer for a second time to adjust the concentration of each sample to 1000 ng/μL. Recombinant DNase I(Takara, D2270A)was served for removal of residual genomic DNA fragments that would interrupt definition of semi-quantitative PCR. To screen the primers, cDNA samples of 2-year after inoculation, the 3-year after inoculation, and healthy controls were mixed separately as templates. Primers with good dissociation curve, amplification curve and without nonspecific amplification were selected for all samples. PCR products were recovered (BioTeke, DP1602) and qualified (Agilent Model 2100 Bioanalyzer) for the 1:10 dilution series to construct the standard curve of each gene.

The 18S rRNA gene was used as an internal control. Primers for 15 selected differentially genes designed using Primer Premier 5 were showed in S1 Table. Programs set for conversional PCR was an initial 5 minute denaturation at 95°C followed by 30 cycles of thermocycling (95°C, 30s; 55°C, 30s; 72°C, 30s), then extended at 72°C for 7 min. Amplifications were carried out in a BIO-RAD C1000 Thermal Cycler. Quantitative PCR (qPCR) was carried out in the Bio-Rad CFX Connect Real-Time System with SYBR Premix Ex *Taq* (Ti RNaseH, Plus) (DRR820B). Standard curve, melting curve, amplification curve, and Ct(CP), Ct(SDM), Qty(CP), and Qty(SDM) were collected for result analyses. Six replicates were used for each sample (three biological replicates × two technical replicates). The relative quantification of target gene transcripts was determined by the comparative CT method 2^−ΔΔCT^ [16], where ΔΔCT = (CT, Target–CT, 18S rRNA)_HLB_−(CT, Target–CT, 18S rRNA)_Control_.

### Data analysis

Expression signals were normalized by Robust Multichip Analysis (RMA) approach[17]. Comparison analysis was performed by the signal ration of treated sample and control sample to identify the differentially expressed genes (DEGs). Genes with ratio of ≤0.5 were considered down-regulated DEGs, while genes with ratio of ≥2 were up-regulated DEGs. The FDR-adjusted significance level cutoff was set at 0.05 for overrepresentation determination. The manual functional analysis was referred to the system of Adams, Kerlavage [18]. Functional enrichment/overrepresentation analysis was carried out using the agriGO database (http://bioinfo.cau.edu.cn/agriGO/) [19].

### Lipoxygenase activity determination

‘*Ca*. L. asiaticus’ infected citrus leaves and healthy leaves collected every two months during one year (From May, 2013) were used for lipoxygenase (LOX) determination. The activity of LOX was detected according to Chen, Xu [20]. Briefly, every two gram of leaf tissue was grinded in 10 mL cold phosphate buffer (50 mmol/L, pH 7.0) with individual mortar and pestle on ice. Liquid supernatant was collected after centrifugation at 15000 g for 15 min for LOX activity detection. The hydroperoxy lipid product of the reaction contains a conjugated diene which absorbs strongly at 234 nm. The reaction was carried out in a solution containing 25μL Linoleic acid sodium salt (25 mmol/L), 2.775 mL acetic buffer (100 mmol/L, pH 5.5) in a final volume of 3 mL. By measuring product formation through the change in absorbance at this wavelength, the quantitative assay of LOX can thus be determined.

### Light microscopy

Mature leaves, all in similar size, with typical mottling and Znic-deficiency-like symptoms were collected from mandarin (*Citrus reticulate* Blanco) trees. Leaves from healthy trees were collected as control. Quantitive real time PCR was conducted to confirm the present of ‘*Ca*. L. asiaticus’. Around 5 mm midribs of each leaf sample were transferred into FAA solution (Volume ratio of 70% ethanol, acetic acid, and 37% formaldehyde was 90: 5: 5) for 48 h. After fixative treatment, samples were gone through paraffin in sections workflow: dehydration, clearing, waxfilling, embedding, and sectioning. Sections were strained with 0.5% dye Fast Green and 1% dye Safranine. Light micrographs were scanned using a light micrograph (Carl Zeiss, West Germany) with an attached camera.

## Results and Discussion

### Host metabolisms were affected by ‘*Ca*. L. asiaticus’ infection

**Fig 2:**
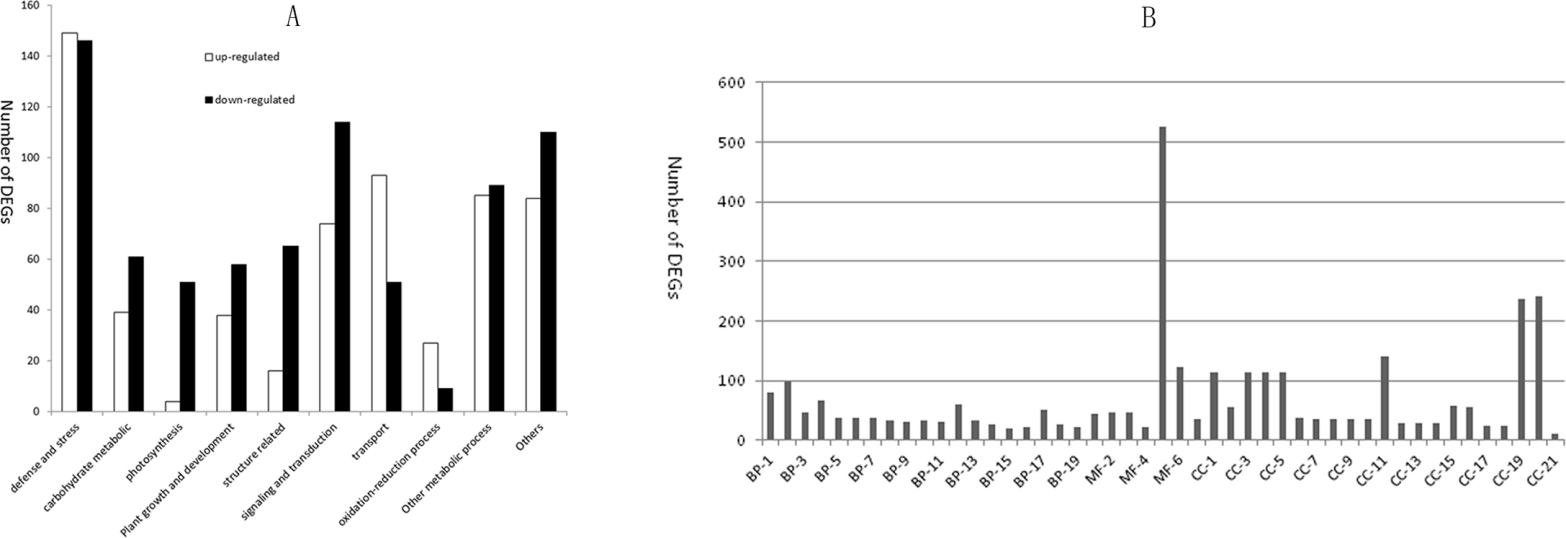
Functional categories and Gene ontology classification of all differentially expressed genes in response to infection with‘*Candidatus* Liberibacter asiaticus’. A, Classification of specifically expressed genes into functional categories in response to ‘*Ca*. L. asiaticus’. B, Gene ontology classification of all differentially expressed genes. DEGs, differentially expressed genes; BP-1, cellular nitrogen compound metabolic process; BP-2, carbohydrate metabolic process; BP-3, polysaccharide metabolic process; BP-4, cellular carbohydrate metabolic process; BP-5, cellular polysaccharide metabolic process; BP-6, cellular glucan metabolic process; BP-7, glucan metabolic process; BP-8, starch metabolic process; BP-9, sucrose metabolic process; BP-10, oligosaccharide metabolic process; BP-11, disaccharide metabolic process; BP-12, electron transport; BP-13, glycoside metabolic process; BP-14, photosynthesis; BP-15, pigment biosynthetic process; BP-16, pigment metabolic process; BP-17, secondary metabolic process; BP-18, phenylpropanoid metabolic process; BP-19, heterocycle biosynthetic process; MF-1, hydrolase activity, hydrolyzing O-glycosyl compounds; MF-2, hydrolase activity, acting on glycosyl bonds; MF-3, transferase activity, transferring glycosyl groups; MF-4, oxidoreductase activity, acting on paired donors, with incorporation or reduction of molecular oxygen; MF-5, catalytic activity; MF-6, oxidoreductase activity; MF-7, transferase activity, transferring hexosyl groups; CC-1, membrane-bounded vesicle; CC-2, thylakoid; CC-3, cytoplasmic membrane-bounded vesicle; CC-4, vesicle; CC-5, cytoplasmic vesicle; CC-6, thylakoid part; CC-7, chloroplast thylakoid; CC-8, organelle subcompartment; CC-9, plastid thylakoid; CC-10, photosynthetic membrane; CC-11, chloroplast; CC-12, cell wall; CC-13, external encapsulating structure; CC-14, thylakoid membrane; CC-15, plastid part; CC-16, chloroplast part; CC-17, chloroplast thylakoid membrane; CC-18, plastid thylakoid membrane; CC-19, plastid; CC-20, membrane; CC-21, photosystem.

Of the 2,017 significantly regulated DEGs, 938 (46.5%) were up-regulated, and 1,080 (53.5%) were down-regulated in infected Jiaogan (All Affymatrix data was submitted to GenBank with GEO accession numbers of GSE67376, http://www.ncbi.nlm.nih.gov/geo/query/acc.cgi?acc=GSE67376). The 1,364 genes affected by ‘*Ca*. L. asiaticus’ in the late stage of infection having known functions were related to plant defense/stress response (23.6%), carbohydrate (sugar/starch/xylan) metabolism (7.9%), photosynthesis (4.4%), plant growth and root/steam/leaf/flower/seed development (7.9%), structure (cell wall metabolism/nucleus organization/ribosomal protein/fiber protein/membrane protein/vacuolar protein) (6.4%), signaling transduction/translation factors (14.5%), DNA/chromatin/nucleotide/ADP/ion/protein/carbohydrate binding (3.9%), sugar/protein/lipid/ion/oligopeptide/dipeptide/tripeptide/amino acid/ATP/ADP/bilirubin/carbohydrate/drug/electron transport (11.9%), oxidation-reduction process (3.2%) with oxidoreductase activity, other metabolic processes (12.8%), and others (10.6%). The expression pattern (up- or down-regulation) of these classified DEGs in the HLB infected trees are shown in Fig 2A. Genes related to photosynthesis and structure were mostly down-regulated by the ‘*Ca*. L. asiaticus’ infection, whereas genes associated with the oxidation-reduction process and transport were mostly up-regulated.

Gene ontology (GO) assignments were used to classify the functions of the DEGs (S2, S3, and S4 Tables), which were assigned to 16 functional groups in the ‘biological processes’ main category, 21 groups in ‘cellular components’ and 7 groups in molecular function’ (Fig 2B). The majority of the GO annotations were positioned in the following categories: photosynthesis, electron transport and the metabolic processes of nitrogen compounds, carbohydrates, polysaccharides, glucans, starch, sucrose, oligosaccharides, disaccharides, glycosides, pigments, and phenylpropanoids and heterocycles.

The significant up-regulation (21.25-fold) of a gene coding the glutamic acid-rich protein cNBL1700, which may be related to pathogenesis [21], was observed. In the interaction with ‘*Ca*. L. asiaticus’, citrus expressed some defense-related genes in response to the stress. Two thi1 genes (Cit.465.1 and Cit.377.1), which may be involved in bacterial-induced DNA damage tolerance in plant cells [22], were up-regulated more than 5 times. Some heat shock protein genes, involved in the regulation of transcription were highly up-regulated, more than 10-fold. The stress related HSP genes were also mentioned in previous studies (induced in [3], but down-regulated in [9]), which functions in plant growth and development [23–27]. However, the expression levels of some DnaJ/Hsp genes were highly depressed.

### ‘*Ca*. L. asiaticus’ depressed the photosynthesis process of mandarin

The photosynthesis process was depressed as a result of HLB-induced incomplete coloring (S5 Table). Photosynthesis-related genes showed opposite expression patterns in leaves (mainly down-regulated) and fruit (mainly up-regulated) in the previous studies. Among the 55 genes related to the photosynthetic process, only 4 (Cit.3817.1, Cit.3817.1, Cit.7266.1 and Cit.17826.1) had increased expression levels after two years of ‘*Ca*. L. asiaticus’ infection. HLB interrupts the citrus metabolic system and causes complete (pale yellowing) or incomplete (blotchy mottle) coloring symptoms on leaves. HLB-infected samples collected in this study displayed typical blotching symptoms. Thus, it is not surprising that photosynthesis-related proteins showed the greatest decrease in response to ‘*Ca*. L. asiaticus’ infection. This is consistent with results from most previous studies that focused on HLB-affected leaves. Most DEGs in this category were photosystem II factors. Seven genes coding light-harvesting chlorophyll a/b binding proteins, including Lil3, cab-11, CP24 and CP29, were repressed in HLB samples compared to healthy samples. The chl a/b-binding protein plays an essential role in chlorophyll and tocopherol biosynthesis reactions in photosystem II [28]. Genes coding chlorophyll a oxygenase, lipoic acid synthase, oxygen-evolving enhancer protein, or Ribulose bisphosphate carboxylase/oxygenase (Rubisco) activase, were down-regulated. Chlorophyll a oxygenase is involved in chlorophyll b formation from chlorophyll a [29]. Additionally, we observed a significant decrease in the accumulation of some subunits of the photosynthetic system in the HLB leaves, including the cytochrome b6f complex subunit (Cit.9493.1 and Cit.40088.1), the photosystem I-N subunit (Cit.8714.1 and Cit.30551.1), oxygen-evolving enhancer protein 2 (Cit.24174.1) and magnesium-chelatase subunit chlI (Cit.21132.1). Violaxanthin de-epoxidase was reported to be involved in the xanthophyll cycle under stress [30]. It is consistent with results from a previous study by Nwugo [9] that Rubisco activase coding genes were repressed. Rubisco activase is a catalytic chaperone that affects photosynthetic induction, nucleotides and sugar phosphates [31, 32]. Photosynthesis is central to all aspects of plant biology. The strong reduction in photosynthesis may relate to the symptoms of the diseased leaves.

### HLB induced the synthesis of starch and sucrose in leaves

Photosynthesis in plants directly influences the carbohydrate metabolic product. Numerous genes with GO terms from the carbohydrate metabolic process (starch anabolism, sucrose, cellulose catabolic process, allantoin catabolic process and sugar metabolism) were differentially expressed in response to HLB (S6 Table). The β-amylase and β-mannosidase enzymes are important for starch degradation [33], while starch branching enzyme, starch phosphorylase type H, granule-bound starch synthase, UDP-glucosyl transferase, UDP-glucose dehydrogenase and isoamylase [34]were related to starch synthesis. Most down-regulated DEGs in this study that were categorized in carbohydrate metabolic process functioned as degrading enzymes. The down-regulated DEGs that had been identified in previous studies were four β-amylase genes (coding an important enzyme in starch degradation [35]), alcohol acyl transferase genes and glycosyl transferase family 8 genes involved in sugar metabolism. The up-regulated genes, coding ADP-glucose pyrophosphorylase, α-amylase, β-glucosidase, glycosyl hydrolase family 38, granule-bound starch synthase, starch branching enzyme, starch phosphorylase type H, UDP-glucuronosyltransferase and starch synthase, had been identified in previous studies on HLB-infected leaves [3, 5, 6] but not on HLB-infected fruits [7]. Five of the differentially expressed β-glucosidase genes were over-expressed (3 by more than 18-fold) in HLB-affected leaf tissues. The genes of alcohol acyl transferase, alcohol dehydrogenase 2, glycerol-3-phosphate dehydrogenase, glycosyl hydrolase family protein, raffinose synthase, fructose-bisphosphate aldolase and trehalose-6-phosphate phosphatase were repressed, as previously reported [5, 9]. However, fructose-bisphosphate aldolase, β-xylosidase, glycosyl hydrolase family protein, β-xylosidase, and β-1,3-glucanase genes were up-regulated by HLB in the study of Albrecht and Bowman [3, 6] but showed no regular pattern at this extremely late infection stage.

Several DEGs that were classified in the carbohydrate metabolic process had not been previously reported in HLB infections. The depressed DEGs were mannosidase genes, xyloglucan-specific fungal endoglucanase inhibitor genes, ribose 5-phosphate isomerase, mannose-6-phosphate isomerase genes and glucan 1,3-β-glucosidase genes. The DEGs specially induced at this extremely late stage included PG1, β-fructofuranosidase, bifunctional lysine-ketoglutarate reductase, glucosyltransferase, exocellular acid invertase 1, limonoid UDP-glucosyltransferase, β-galactosidase and hexose transport protein HEX6. As a cellulose catabolic process gene, β-galactosidase may also play a role during abscission, and growth and development processes in flowers and fruitlets [36]. An exocellular acid invertase 1 with fructan β-fructosidase activity [33] and a limonoid UDP-glucosyltransferase with sinapate 1-glucosyltransferase activity had expression levels that were increased 6.5 and 5.4 times after HLB infection. Limonoid UDP-glucosyltransferase is supposed to be highly associated with the sweet or sour taste of citrus fruits [37]. The up-regulation of key starch synthases and sugar biosynthetic genes, together with the down-regulation of related degrading enzymes, likely leads to the accumulation of starch in HLB-affected leaves and the sour taste in the affected fruits.

### Plant growth and development was influenced by ‘*Ca*. L. asiaticus’ infection

Photosynthesis is an important biological process in plants because it provides energy for plant growth. About 7.9% of the DEGs induced by ‘*Ca*. L. asiaticus’ at the very late stage were classified in the ‘plant growth and development’ category. All 4 differentially expressed flavonol synthase genes were induced. The flavonol synthase genes from the ‘Satsuma’ mandarin were differentially regulated in the fruit developmental stage in a tissue-specific manner [38]. At 25 months after infection, genes encoding bifunctional nuclease, CCAAT-box-binding transcription factor-related protein, GGPP synthase, GPI-anchored protein, NAC domain protein NAC2 and SRG1 protein were highly induced, while genes of a TCP family transcription factor, two rapid alkalinization factor 2s, one growth-regulating factor, two rapid specific-tissue protein 2s, four arabinogalactan proteins and two ent-kaurene oxidases were significantly suppressed. The CCT (for CONSTANS, CONSTANS-LIKE and TOC1) domain is involved in photoperiodic flowering, light signaling and circadian rhythm regulatory processes in *Arabidopsis thaliana*. It is similar to the yeast HEME ACTIVATOR PROTEIN2 (HAP2) that binds to CCAAT boxes in eukaryotic promoters [39]. Levels of bifunctional nuclease 1 mRNA were significantly different in different plant tissues and were induced during leaf and stem senescence [40]. Similarly, the up-regulated GGPP synthase functioned in the leaf senescence in *Arabidopsis* [41]. The GPI-anchored protein was involved in the pollen tube growth process [42]. SRG1 proteins involved in embryo development ending in seed dormancy or organ senescence were also induced in sweet orange infected by ‘‘*Ca*. L. asiaticus” [4]. Two GO results for the TCP family transcription factor are the negative regulation of leaf senescence (GO:1900056) and root development (GO:0048364). Additionally, the plant growth and development-related arabinogalactan proteins were reported to be repressed in ‘Valencia’ orange leaves responding to the HLB pathogen [3]. The nt-kaurene oxidase is a gibberellin biosynthesis enzyme [43] that functions in the gibberellin biosynthesis pathway, and subsequently influences plant growth. Rapid alkalinization factors were reported in the brassinosteroid-mediated signaling pathway that negatively regulates plant growth. No highly induced or suppressed genes were found, which may due to the long time interaction of citrus plants with the pathogen that may have allowed the host to develop a maintenance level towards this stress.

### Plant cell organelles were damaged by the 2 year long HLB infection

Also noticeable among the highly down-regulated genes were those for ribosomal proteins, membrane proteins, thioredoxins and vacuolar proteins. In total, 81 DEGs were classified as structural proteins. Most (80.2%) of the structures were depressed in this very late stage of infection (S7 Table). Among the DEGs, the ribosomal proteins were especially prominent (24 of the 28 were down-regulated). However, the ribosomal proteins involved in protein metabolism were up-regulated in the study of Albrecht and Bowman [6]. Transcripts coding for hydroxyproline-rich glycoproteins were also noticeably down-regulated. Thioredoxins, components of the plant cell redox regulatory system that function as antioxidants in ROS scavenging, were up-regulated in HLB-affected sweet orange fruit [8] but down-regulated in lemon leaves [10].

**Fig 3:**
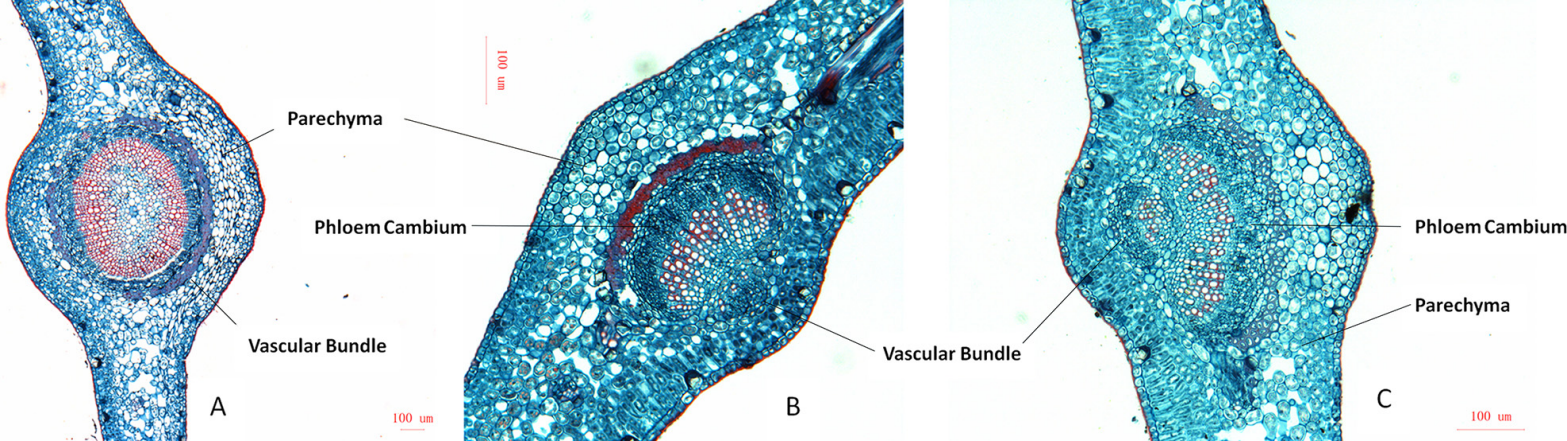
Anatomical analysis (Fast Green and Safranine O) of midrib phloem tissues of the huanglongbing (HLB) affected and healthy *Citrus reticulata* Blanco using light microscopy. **A**, midrib of healthy leaf. Vascular bundle is in oval-shaped, all tissues are well developed, the interior of parenchyma cells are relatively clear. **B** and **C**, Huanglongbing infected midribs from leaves with blotchy mottle or Zinc-deficiency-like symptoms. Parenchyma contains more internal substances like starchs, half round of vascular bundle is poor developed and phloem cambium is thickened.

The midribs of HLB-affected and uninfected mandarins were stained and observed with LM (Fig 3). Significant disorder of the cells and collapse of sieve tubes and companion cells were observed. The phloem layer and cambium layer of infected samples were thicker than uninfected midribs. Starch accumulation was more serious in the diseased midribs. These anatomical changes in ‘*Ca*. L. asiaticus’ affected samples were similar but stronger than previous report [5].

### Transcription/signal transduction-related genes were alerted

A total of 82 transcriptional genes or genes with transcription factor activity were differentially regulated after two years of ‘*Ca*. L. asiaticus’ infection (S8 Table). Of these, 46 were down-regulated. The MADS-box protein 4 coding gene (Cit.144.1) was induced by 13-fold. A TAZ zinc finger family protein (Cit.6280.1.S1), with a GO classification as a negative regulator of transcription from the RNA polymerase II promoter, was increased by11.8-fold. However, for the DEGs encoding certain transcription factors, 3 WRKY family, 5 myb family, 10 AP2 domain, 10 bHLH and 3 heat shock, as well as 6 genes annotated as unknown proteins with transcription factor activity and 3 zinc finger (B-box type) family proteins, the expression profiles were not consistent within the groups. Consequently, no conclusions about transcription factor-regulated signaling pathways were drawn, unlike in some studies focusing on earlier HLB infection stages. However, the transcripts of differentially regulated C_2_H_2_-type and GATA-type zinc finger proteins, auxin/indole-3-acetic acid proteins and squamosa promoter-binding proteins were depressed. Whereas, three genes encoding scarecrow transcription factor family proteins, three genes of the bZIP transcription factor family, and two genes encoding dehydration-responsive element binding proteins [44, 45], were all up-regulated.

In addition to the transcription factor genes, another 107 genes encoding proteins involved in the regulation of transcription processes or signal transduction were classified in the signaling category. For these genes, most (69 of 107) were down-regulated. The most prominent groups included 14 of 15 genes encoding unknown proteins, 6 genes for ethylene responsive element binding proteins or ethylene-induced esterases, and 4 dormancy-associated protein genes. The expression of other genes was also suppressed, including two copies of the genes encoding transducin-like, GTP cyclohydrolase, allergenic isoflavone reductase-like protein Bet v 6.0102 and gibberellin-regulated proteins. All three Avr9/Cf-9 rapidly elicited proteins (Cit.6781.1, Cit.15649.1 and Cit.16734.1) were decreased by more than 2.8-fold. Avr9/Cf-9 rapidly elicited proteins are thought to be involved in the ionotropic glutamate receptor signaling pathway and hypersensitive responses in plant cell death, a disease-resistance pattern [46].

The ‘*Ca*. L. asiaticus’ infection induced the transcription of 38 genes in signaling pathways, such as genes encoding 33 kDa secretory protein-related, α-expansin 3, TMKL1 precursor, ionotropic glutamate receptor homolog GLR4, response regulator 6, RESPONSE REGULATOR 9, cyclic nucleotides, calmodulin-regulated ion channel proteins and homogentisate geranylgeranyl transferase. Four no apical meristem (NAM)-like protein genes and 3 NAM family protein genes had increased transcript levels. No regular patterns for the responses of the 8 serine/threonine kinases, 4 isoflavone reductases or 11 protein kinases to ‘*Ca*. L. asiaticus’ were observed.

### Other metabolic processes influenced by HLB

DEGs in this category were related to the biological processes of terpene synthase, lipid metabolism, cell/tissue development and cell wall metabolism, acyl-carrier-protein biosynthesis, asparagine biosynthesis, sterol biosynthesis, fatty acid biosynthesis, isoprenoid biosynthesis, chitin metabolism, Mo-molybdopterin cofactor biosynthesis, flavonol biosynthesis, UMP biosynthesis and other secondary metabolisms. Of these, 85 (48.9%) were depressed during the late stage of ‘*Ca*. L. asiaticus’ infection. Among the down-regulated genes, those encoding 3 acid phosphatases, 6 α- or β-tubulins, 3 early nodulinses, 4 pectate lyases, 7 pectin esterases, and 2 cinnamoyl-CoA reductases were apparent. The expression pattern of the pectin esterase-coding genes was different in fruit. Consistent with the study of Nwugo [9], cinnamoyl-CoA reductase genes and tubulin genes were significantly depressed in response to ‘*Ca*. L. asiaticus’. However, the energy/metabolisms-related genes of the cytosolic aldehyde dehydrogenase and dienelactone hydrolase family proteins/carboxymethylenebutenolidase were not increased in the study by Nwugo [9].

Significantly, levels of transcripts for the two CCR proteins [47, 48], two catechol O-methyltransferases (involved in synthesis of the flavor molecule guaiacol [49, 50]), and two caffeic acid O-methyltransferase IIs, which function in lignin, flavonoids and sinapoyl malate biosynthesis [51, 52], were increased more than 5-fold. Cit.21988.1.S1, which is involved in fatty acid/lipid and amino acid metabolism, was induced 3.4-fold in this study, but was decreased in fruit [8]. The four genes encoding cellulose synthases, related to cell organization and biogenesis, and the UDP-glucose metabolic process, were reported to be depressed in Albrecht and Bowman (3], but were induced here. A fatty acid/lipid and amino acid metabolism gene encoding family II extracellular lipase 3 was highly (7.13-fold) induced here, as well as in fruit [8]. The other 4.5-fold and 7.1-fold up-regulated genes *FPh1*, related to the phospholipase/carboxylesterase family leucoanthocyanidin dioxygenase-like protein [53] and O-methyltransferase family 2 protein, respectively, were also induced by ‘*Ca*. L. asiaticus’ in other studies. The up-regulation of oxidoreductase, 2-oxoglutarate and Fe(II) oxygenase family proteins and lipase class 3 family protein genes, as well as the down-regulation of monogalactosyldiacylglycerol synthase and H+-transporting ATP synthase genes were consistent with previous research on ‘*Ca*. L. asiaticus’[6, 8, 9].

### HLB alteration of the hormone-mediated immune response

Hormone signaling pathways are reported to strongly relate to plant disease progress. A lot of phytohormone related genes were differentially expressed in mandarins plants infected with ‘*Ca*. L. asiaticus’ (S9 Table). The expression of genes encoding three ethylene-responsive element binding proteins, 1-aminocyclopropane-1-carboxylate oxidase, ethylene response factor ERF3b and ethylene-induced esterase, were all down-regulated by the ‘*Ca*. L. asiaticus’ infection. Additionally, all five genes encoding aquaporin proteins had decreased expression levels. Aquaporin PIP1 interacts with PIP2 in ethylene-regulated plant growth [54]. Only a gene encoding an ethylene-forming-enzyme-like dioxygenase-like protein was induced under the long term infection. Consequently, this study suggests that the ethylene pathway was depressed in this study, which is different from previous studies. Reduced transcription was observed for genes encoding a NPR1/NIM1-interacting protein 3, a BTB/POZ domain-containing protein, a S-adenosyl-L-methionine:salicylic acid carboxyl methyltransferase, a UDP-glucose:salicylic acid glucosyltransferase, a catalase, Nine glutathione peroxidase and a Cu/Zn superoxide dismutase that are involved in salicylic acid signaling. Moreover, the transcriptional patterns of salicylic acid-mediated defense response-related proteins, PR5-1, PR1 and PR10A, were different [55]. To conclude, a two year ‘*Ca*. L. asiaticus’ infection triggered a response via both salicylic acid and ethylene pathways.

**Fig 4:**
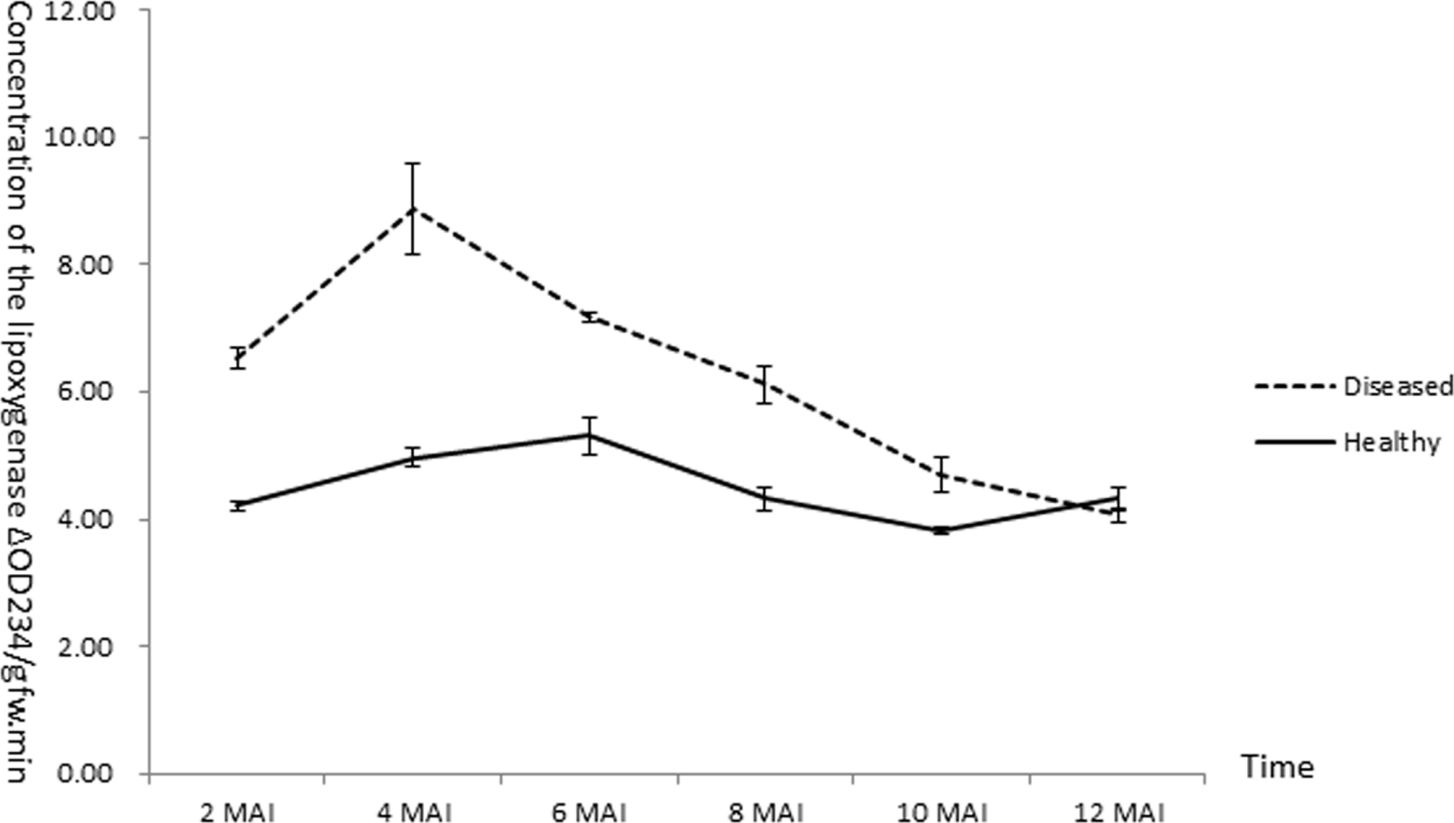
Evaluation of the time course lipoxygenase (LOX) activity in huanglongbing (HLB) affected and healthy *Citrus reticulata* Blanco leaves.

Seventeen Of the 20 DEGs related to the jasmine acid signaling pathway (S9 Table)., including four LOX coding genes, three NAM family protein genes and seven glutathione S-transferase genes were predominately up-regulated compared with in non-infected trees. Three replicates of leaves collected every two months from HLB infected trees and healthy trees were used to determine the LOX activity. The one-year data showed that the concentration of LOX in the diseased trees was relatively higher than that in healthy trees among the year (Fig 4). This is consistent with the array data.

Strikingly, eight genes corresponding to auxin-regulated proteins were down-regulated, while genes encoding auxin-repressing proteins were up-regulated (S9 Table). This indicates that the auxin-regulated pathways were suppressed after 2 years of a ‘*Ca*. L. asiaticus’ infection. Similarly, several genes involved in the signal transduction of gibberellins and abscisic acid were induced while some of them were depressed. A gibberellin-responsive GAST1 homolog was 11.1-fold up-regulated. This gene was predicted to be involved in brassinosteroid, gibberellin and abscisic acid responsive expression by GO analysis.

### Other stress-related genes are differentially regulated by ‘*Ca*. L. asiaticus’

The two year ‘*Ca*. L. asiaticus’ infection up-regulated the expression of 149 plant defense or stress-response genes, including homologs of protein kinase Xa21, SAR related N-hydroxycinnamoyl/benzoyltransferase (4.35-fold), disease resistance protein (also up-regulated in previous studies of HLB-citrus interactions), glutathione S-transferase, EIX receptor 2 [56, 57], hypersensitive-related kinases, like HSR203J [58], abiotic stress-related calmodulin [59] and resistance-related RPP8-like protein. Consistent with some of the previous studies [4, 10], the following genes were up-regulated: a gene coding receptor-like kinase CHRK1 (6.3-fold), five ATPase genes (7.5-, 4.6-, 3-, 2.6- and 2.4-fold), and eight stress/defense response chitinase genes (10.4-, 6.8-, 5.2-, 5.1-, 4.5-, 4.1-, 2.6- and 2.5-fold), involved in cell organization and biogenesis.

Among the genes down-regulated more than 2-fold were enzymes associated with aquaporin (5), aspartyl protease family protein (2), subtilisin-like protease (3), invertase/pectin methylesterase inhibitor (4) [60, 61], leucine rich repeat protein (9 of 11), which was different from in Fan et al. [55, 62], and miraculin-like protein 2 (9 of 10) (induced in[10] and [4]). A gene putatively coding an alanine-glyoxylate aminotransferase, which functions in the rice-blast interaction was 7.3-fold induced [63].

### qRT-PCR validation

**Fig 5:**
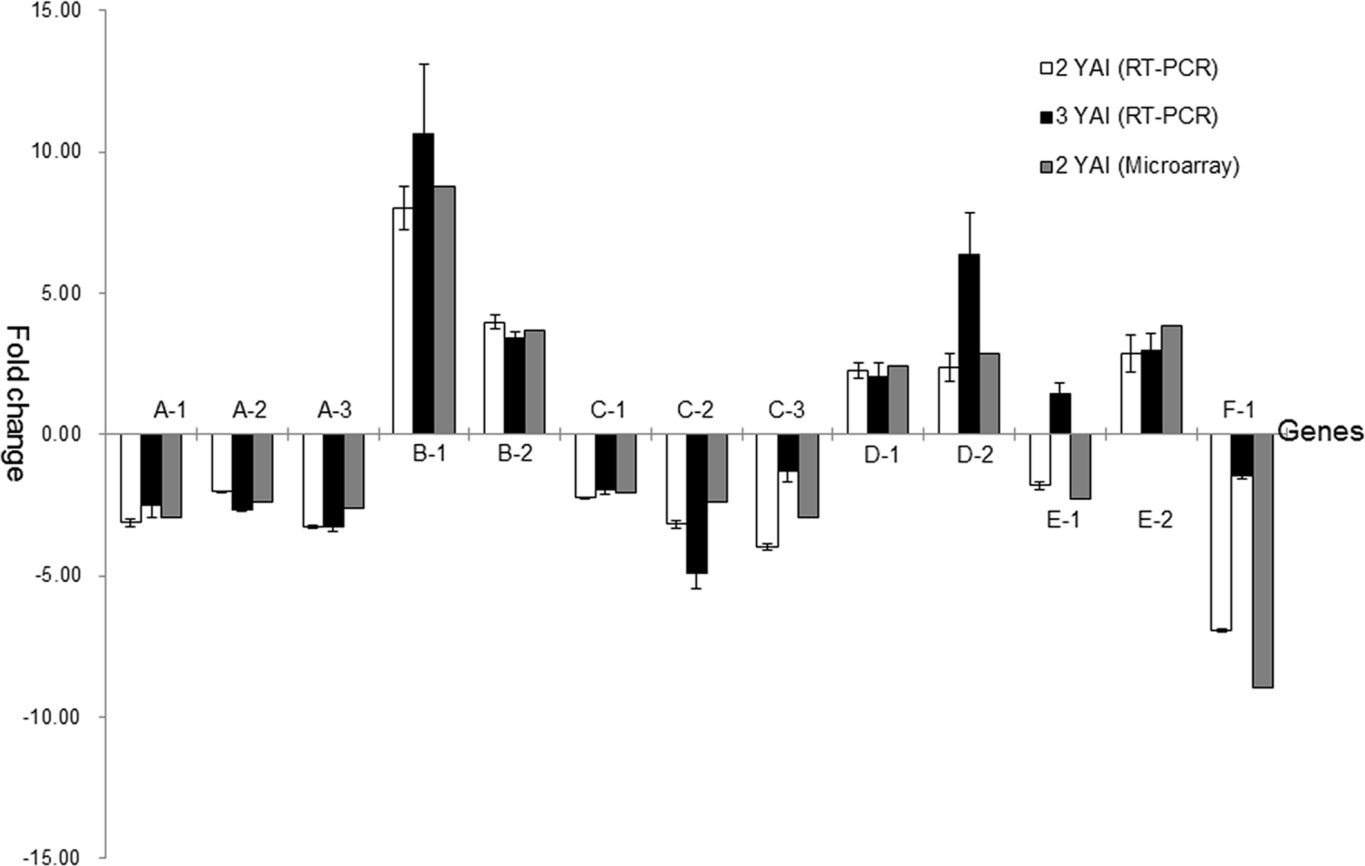
Real time PCR and microarray profiles of 15 selected differentially expressed genes (DEGs). A, ethylene pathway related genes; B, jasmine acid pathway related genes; C, photosynthetic related genes; D, transport related genes; E, starch degradation or synthetic genes; F, plant defense and stress related genes. A-1, ethylene responsive element binding protein; A-2, ethylene response factor ERF3b; A-3, ethylene responsive element binding protein; B-1, no apical meristem (NAM) family protein; B-2, glutathione S-transferase GST 14; C-1, NADPH-protochlorophyllideoxidoreductase; C-2, light-harvesting chlorophyll a/b binding protein; C-3, chlorophyll synthase, putative; D-1, glucose-6-phosphate/phosphate translocator; D-2, glucose-6-phosphate/phosphate-translocator precursor; E-1, beta-amylase; E-2, starch branching enzyme; F-1, leucine rich repeat protein.

To corroborate the microarray data, 15 genes classified in different functional groups were amplified using the quantitative real time PCR method. Samples used were from Jiaogan leaves infected by ‘*Ca*. L. asiaticus’ for 2 or 3 years, and their respective non-infected controls. The gene coding glutamic acid-rich protein cNBL1700, a pathogenesis related gene, which was induced 21.25 times when screened using a microarray, was 15.8±3.47 and 5.13±0.79 times up-regulated after 2 and 3 years, respectively. A chalcone synthase gene, related to plant growth and development, was detected to be highly depressed in the microarray result, and the trends were the same, 5.22±0.02 and 20.01±7.00 fold down-regulated after 2 or 3 years, respectively, for the real time PCR results. The expression profiles of all 15 genes were consistent with the microarray data when using the same samples tae at 2 years after infection (Fig 5). The expression patterns of most genes in the samples taken 3 years after infection were similar to, but stronger than, those taken after 2 years (Fig 5).

## Conclusions

The broad range of DEGs suggests that citrus trees were profoundly disturbed after 2 years of infection with the bacterial disease HLB. Firstly, the photosynthesis process was most dominantly depressed. Consequently, the carbohydrate metabolic process, and plant growth and development were influenced. The ‘*Ca*. L. asiaticus’ infection also down-regulated the transcription of genes encoding structural protein and disrupt the cell of midribs. Thirdly, the ethylene and salicylic acid signaling pathways were depressed while the jasmine acid signaling pathways were induced. Finally, an analysis of differentially-regulated signaling genes and enriched stress-related genes demonstrated that multiple regulatory pathways, including NAM, nectarin 5, tubulin, chitinase CHI1, and miraculin-like protein 2 regulons, are involved in the very late stage of ‘*Ca*. L. asiaticus’ infection. Compared to earlier infections, the metabolic processes of nitrogen compounds, polysaccharides, oligosaccharides, disaccharides, glycosides, pigments, phenylpropanoids and heterocycles were especially activated by ‘*Ca*. L. asiaticus.

## Supporting Information

S1 Table Quantitative RT-PCR primer sets used to determine differential expression between mandarins infected by ‘*Candidatus* Liberibacter asiaticus’ for 2 years and healthy cirrus. (XLS)

S2 Table Gene ontology enrichment result of the differentially expressed genes. P, Biological Process; F, Mollecular Function; C, Cellular Componen.
(XLS)

S3 Table Gene ontology enrichment result of the down regulated genes. F, Mollecular Function.
(XLS)

S4 Table Gene ontology enrichment result of the up-regulated genes. P, Biological Process; F, Mollecular Function; C, Cellular Componen.
(XLS)

S5 Table Functional classification of photosynthetic process related genes with differential expression (2-fold) in mandarins plants infected with ‘*Ca*. L. asiaticus’ compared with non-infected plants 25 months after inoculation. (XLS)

S6 Table Huanglongbing regulation of genes related to carbohydrate metabolic. (XLS)

S7 Table Huanglongbing regulation structure protein genes. (XLS)

S8 Table Huanglongbing regulation of transcriptional factors and signaling genes. (XLS)

S9 Table The plant hormone related genes regulated by 25 months’ Huanglongbing infection. SA, Salicylic acid; JA, Jasmine acid; GI, Giberellin; ET, Ethylene; CT, Cytokinin; BR, Brassinosteroid; AU, Auxin; IAA, indole-3-acetic acid; ABA, Abscisic acid.
(XLS)
